# OPN inhibits autophagy through CD44, integrin and the MAPK pathway in osteoarthritic chondrocytes

**DOI:** 10.3389/fendo.2022.919366

**Published:** 2022-08-12

**Authors:** Rui-Jun Bai, Di Liu, Yu-Sheng Li, Jian Tian, Deng-Jie Yu, Heng-Zhen Li, Fang-Jie Zhang

**Affiliations:** ^1^ Department of Orthopaedics, Xiangya Hospital, Central South University, Changsha, China; ^2^ National Clinical Research Center for Geriatric Disorders, Xiangya Hospital, Central South University, Changsha, China; ^3^ Department of Emergency Medicine, Xiangya Hospital, Central South University, Changsha, China

**Keywords:** autophagy, chondrocyte, integrin, CD44, osteoarthritis, osteopontin

## Abstract

**Background:**

To investigate whether osteopontin (OPN) affects autophagy in human osteoarthritic chondrocytes and determine the roles of CD44, αvβ3 integrin and the Mitogen-activated protein kinase (MAPK) pathway in this progress.

**Methods:**

First, we compared the autophagy levels in the human osteoarthritis (OA) and normal cartilage, then, we cultured human OA chondrocytes *in vitro* and treated cells with recombinant human OPN (rhOPN) to determine autophagy changes. Next, the anti-CD44 and anti-CD51/61 monoclonal antibodies (Abs) or isotype IgG were used to determine the possible role of CD44 and αvβ3 integrin; subsequently, an inhibitor of the ERK MAPK pathway was used to investigate the role of ERK MAPK. Western blotting was used to measure the Beclin1, LC3 II and MAPK proteins expressions, mRFP-GFP-LC3 confocal imaging and transmission electron microscopy were also used to detect the autophagy levels. Cell Counting Kit-8 (CCK-8) was used to assay the proliferation and activity of chondrocytes.

**Results:**

The LC3 protein was greatly decreased in OA cartilage compared to normal cartilage, and OPN suppressed the autophagy activity in chondrocytes *in vitro*. Blocking experiments with anti-CD44 and anti-CD51/61 Abs indicated that OPN could suppress the expression of LC3II and Beclin1 through αvβ3 integrin and CD44. Our results also indicated that the ratio of p-ERK/ERK but not p-P38/P38 and p-JNK/JNK was increased after the rhOPN treatment. The ERK inhibitor inhibited the activity of OPN in the suppression of autophagy, and the CCK-8 results showed that rhOPN could promote chondrocyte proliferation.

**Conclusion:**

OPN inhibited chondrocyte autophagy through CD44 and αvβ3 integrin receptors and *via* the ERK MAPK signaling pathway.

## Background

Osteoarthritis (OA) is the most common joint disease of the elderly and dysfunction of the lower limbs, and it can affect the whole joint, thus leading to meniscal degeneration, inflammation and fibrosis of the synovial membrane and infrapatellar fat pad; however, OA is primarily characterized by the progressive breakdown of articular cartilage (1). Radiographic evidence of OA mainly occurs in the elderly individuals over the age of 65, with approximately 80% of the patients over the age of 75 (2). The pathogenesis of OA involves a variety of factors, including mechanical factors, genetic factors and age-related factors, with age representing the main risk factor for OA (3, 4). Chondrocytes have the ability to respond according to the structural changes around the cartilage matrix, although the capacity of adult chondrocytes to regenerate normal cartilage matrix architecture is restricted and decreases with age (5, 6). Therefore, maintaining chondrocytes in a healthy state seems to be a significant factor for maintaining intact cartilage and preventing its degeneration (5, 7). However, the etiology of the disease remains poorly understood.

Autophagy is an important mechanism by which cells self-eliminate nonfunctional organelles and macromolecules, especially in the case of increased catabolic metabolism and nutritional stress (7). Autophagy plays a protective or homeostatic role in normal cartilage (8). The occurrence and development of aging-related diseases are closely related to defects in autophagy, and recent studies have shown that this process is relevant to the damage and degeneration of chondrocytes (9). Autophagy activity is decreased in human OA and age-related or surgically induced animal model OA, and the expression of autophagy-related proteins is decreased (8). Using rapamycin to activate LC3 could reduce the lesion size and severity of cartilage degeneration in experimental OA and may be an effective therapeutic approach for OA (10).

Osteopontin (OPN) is a multifunctional phosphoprotein with a molecular weight of 44-77 kDa that is synthesized and secreted by many tissues and cells, including chondrocytes and synovial cells (11, 12). OPN mRNA is increased in human OA cartilage compared with normal cartilage (11). Furthermore, OPN in plasma, synovial fluid and articular cartilage is closely related to the progression of joint degeneration and may represent a biochemical indicator of disease progression and severity (13–15). OPN has several cell surface receptors, including integrin and CD44 (16, 17). Both integrin and CD44 play important roles in OA progression (16, 18, 19), and CD44 in articular cartilage is related to the degree of OA joint damage (20). However, the roles of OPN and its receptors in the pathological processes of knee OA are unknown.

Mitogen-activated protein kinase (MAPK) is the necessary upstream signaling pathway in aggrecanase and matrix metalloproteinase (MMP)-mediated articular cartilage degradation and plays a key role in regulating the OA process (21). The MAPK pathway plays a negative regulatory role in autophagy at the early stage of OA (22), and regulation of the MAPK signaling pathway has an effect on autophagy levels in articular cartilage (23).

To date, the pathways involving integrin/CD44 and MAPK in autophagy mediated by OPN in OA have not been determined. In the present study, we attempted to clarify the complex mechanism by which OPN regulates chondrocyte autophagy.

## Materials and methods

### Ethics approval

The study protocol was approved by the Institutional Ethics Committee (No. 201503192) of Xiangya Hospital Central South University, and the patients provided written informed consent to participate in this study.

### Cultures of chondrocytes

The chondrocytes culture protocol complied with our previous study (24). Samples of osteoarthritic cartilage were isolated from 10 knee OA patients who underwent total knee replacement, and the knee OA diagnosis was performed according to the guidelines for the diagnosis and treatment of osteoarthritis in China (25). Normal samples were obtained from the knees of 3 age-matched traumatic amputation patients, without any history of OA or other joint diseases, including rheumatoid arthritis and septic arthritis. Normal cartilage and OA cartilage were confirmed using the modified Outerbridge classification (26, 27). The cartilage was first washed with phosphate-buffered saline (PBS) twice, and then the cartilage tissue was chopped into 1-5 mm^3^ slices by a scalpel. The small cartilage pieces were added to a test tube containing 5-8 ml 0.2% collagenase II (Sigma-Aldrich, St. Louis, MO, USA), and the tube was then placed in a constant temperature shaker for digestion for 12-16 h at 37 °C. The progress of digestion was terminated by the addition of 8-10 ml of Dulbecco’s modified Eagle’s medium/F12 (DMEM/F12; HyClone, Logan, UT, USA). Then, the released cell pellet was aspirated from the bottom of the test tube, centrifuged at 1000 rpm for 5 min, and transferred to a culture flask. Each culture well was contained with 5 ml DMEM/F12 with 10% fetal bovine serum (FBS; Gibco, Grand Island, NY, USA) and 1% penicillin/streptomycin solution (Gibco) and was incubated for 24 h at 37°C with 5% CO_2_. Subsequently, the DMEM/F12 was changed every 3 days before trypsinization for the next experiment, and the nonadherent cells were washed out with PBS when the growth medium was changed. The passage 1 chondrocytes were identified using a type II collagen antibody (1:1000, Abcam, Cambridge, UK) as our previous study with immunohistochemistry staining (28) and used for all experiments.

### Cell treatment

The passage 1 chondrocytes were plated in 6-well culture plates at 5×10^5^/well and serum-starved for 24 h in DMEM/F12 containing 1% fetal bovine serum (FBS; Gibco, Grand Island, NY, USA) for 24 h. First, to determine whether OPN had an effect on autophagy, the human OPN (rhOPN) group was treated with 100 ng/ml rhOPN (1433-OP; R&D Systems, Minneapolis, MN, USA) for 48 h, and the control group was unstimulated and untreated, the rhOPN intervene concentration and time was according to our previous results (29).To induce autophagy activity, the cells were treated with rapamycin (10 μmol/L; Cell Signaling Technology, Boston, MA, USA). Next, blocking experiments were performed to determine the possible role of CD44 and αvβ3 integrin. Chondrocytes were treated with mouse anti-CD44 monoclonal antibody (20 µg/ml; BD Biosciences, Franklin, New Jersey, USA) or isotype control IgG2 (20 µg/ml BD Biosciences, Franklin, New Jersey, USA) and anti-CD51/61 monoclonal antibody (20 µg/ml; BD Biosciences, Franklin, New Jersey, USA) to block the binding of OPN-αvβ3 integrin or isotype control IgG1 (20 µg/ml BD Biosciences, Franklin, New Jersey, USA) 1 h prior to the rhOPN treatment, which was performed for 48h, the intervention reagent concentration were according to the previous study (30–32).

Subsequently, to investigate the role of MAPK in the process, special inhibitors of ERK MAPK (FR180204 10 µmol/L, Beyotime, China) were used 1 h prior to rhOPN treatment for 48 h according to previous experimental results.

### mRFP-GFP-LC3 lentivirus transfer and laser confocal imaging

Chondrocytes were seeded into glass bottom cell culture dishes (nest, No. 801002) at 2×10^5^/dish. The next day, the mRFP-GFP-LC3 lentivirus (HanBio, shanghai, China) was transferred at a multiplicity of infection (MOI) of 100. After 8 h, the virus-containing medium was changed to DMEM/F12 containing 10% fetal bovine serum, and the cells were treated as previously described. After 48 h, the mRFP-GFP-LC3 puncta were visualized using a Leica laser confocal microscope. mRFP was used to label and track LC3. The weakening of GFP can indicate the fusion of lysosomes and autophagosomes to form autophagolysosomes because GFP fluorescent protein was sensitive to acid. When autophagosomes and lysosomes were fused, GFP fluorescence was quenched, and only red fluorescence could be detected at this time. Under microscope imaging, the strength of the autophagy flux can be clearly observed by counting the different color spots. The red spots (RFP) represented autophagolysosomes, and the yellow spots (merge) represented autophagosomes (RFP+GFP), the process, used concentration and results interpretation were according to the manufacturer’s instructions.

### Western blot

The western blot protocol complied with our previous study (24). The proteins extracted from cartilage tissue and cultured chondrocytes were tested. The protein extracted from cartilage tissue in liquid nitrogen immediately after the cartilage tissue was washed and ground into small sections. For the western blot analysis of cultured chondrocytes, 100μl/well SDS containing protease inhibitor (100:1) was used to lyse chondrocytes, and a BCA protein assay kit (Thermo Fisher Scientific, Boston, MA, USA) was used to measure the protein concentration of the lysate. Forty micrograms of protein were used for subsequent experiments, such as electrophoresis. Twelve percent SDS-polyacrylamide gel electrophoresis (SDS-PAGE) (Genescript, Piscataway, USA) was performed to separate the proteins at 80 v for 30 min and then 120 v for 60 min, and they were then transferred to polyvinylidenedifluoride (PVDF) membranes with 300mA, 75min. The PVDF membrane was sealed in skimmed milk powder for 1 h and incubated overnight at 4 °C with beclin1 (1:1000, Cell Signaling Technology, Boston, MA, USA), LC3 (1:1000, Cell Signaling Technology, Boston, MA, USA), p38(1:1000, Abcam, Cambridge, UK), p-p38(1:1000,Abcam, Cambridge, UK), extracellular signal-regulated kinase (ERK) (1:1000, Abcam, Cambridge, UK), p-ERK(1:1000, Abcam, Cambridge, UK), Jun Nterminal kinase (JNK) (1:1000, Abcam, Cambridge, UK), p-JNK(1:1000, Abcam, Cambridge, UK), and primary antibody dilution buffer and then incubated with horseradish peroxidase (HRP)-conjugated anti-rabbit IgG (1:2000) for 1 h at room temperature. β-actin and GAPDH were used as housekeeping genes. After that, enhanced chemiluminescence (ECL) (NCM Biotech, Suzhou, China) was performed to develop the membranes, Bio-Rad ChemiDoc-XRS with Image Lab software (BioRad, Richmond, CA, USA) was used to expose and qualify the membranes, and western blot experiments were repeated 3 times.

### Transmission electron microscopy

The chondrocytes were harvested and fixed in 2.5% glutaraldehyde overnight, then fixed in 1% osmium tetroxide for 1h at room temperature, washed with PBS several times, subsequently dehydrated with different concentrations of acetone for 10 min each time, finally embedded in 1:1 acetone/embedding resin at room temperature for 12 h. The cell pellets were cut with an ultramicrotome (Leica, Germany) into 50–80 nm sections and further stained with uranyl acetate and lead citrate. The autolysosomes were observed with the transmission electron microscope (HITACHI, Japan).

### Cell proliferation assay

The procedure was according to the manufacturer’s instructions. Chondrocytes were seeded at 10^4^/well in a 96-well plate, and five wells were measured for each same treatment. After 48 h of treatment, a Cell Counting Kit-8 (CCK-8) assay (Beyotime,China) was used to determine cell proliferation. Then, 90 μl DMEM/F12 culture medium whit 10 μl CCK-8 solution was added to each well and incubated for an additional 2 h. Absorbance was detected at a wavelength of 450 nm by a microplate reader (BioRad, Richmond, CA, USA). For each intervention, only the data from middle three wells were taken for analysis.

### Statistical analysis

All statistical calculations were performed using GraphPad Prism 6.0 (GraphPad Software, Inc., La Jolla, CA, USA). Data are expressed as the mean ± standard error of the mean. The statistical analysis of the differences between two experimental groups was performed using Student’s t test. Differences among groups were determined by the one-way ANOVA; to compare the difference of each group, the Bonferroni test was used. A *P* value less than 0.05 was considered as statistically significant.

## Results

### RhOPN inhibit autophagy in chondrocyte *in vitro*

**Figure 1:**
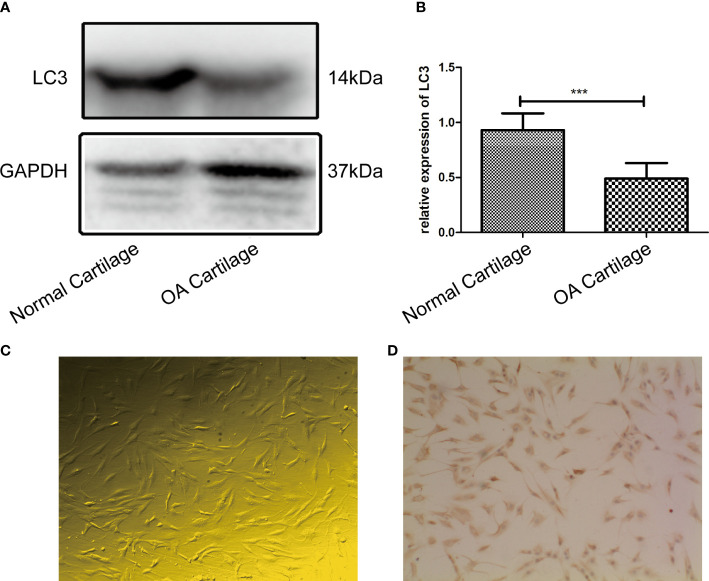
**(A)** LC3 extracted from the OA cartilage (n = 3) was significantly decreased compared to that extracted from normal cartilage (n = 3) by western blot analysis; **(B)** Difference in expression of LC3 between the two groups was statistically significant (P = 0.0007, P < 0.05). **(C)** The passage 1 OA chondrocytes were small, most of them had irregular shapes such as triangles or polygons. Cytoplasmic endoplasmic reticulum and Golgi apparatus were abundant(100×). **(D)** Immunohistochemistry staining revealed that chondrocytes expressed high levels of type II collagen, and the positive substance was brown, mainly distributed in the cytoplasm and extracellular matrix of OA chondrocytes (100×). *** indicates P<0.001 for comparisons between the two groups.

The LC3 protein was significantly decreased in OA cartilage compared to normal cartilage, and the difference between the two groups was statistically significant (*P*=0.0007, *P*<0.05), as shown in **Figures 1A, B**, OA chondrocytes (passage 1) exhibited irregular shapes such as triangles or polygons (**Figure 1C**). Immunocytochemistry staining revealed the positive reaction of type II collagen antibody in the majority of chondrocytes (**Figure 1D**).

**Figure 2:**
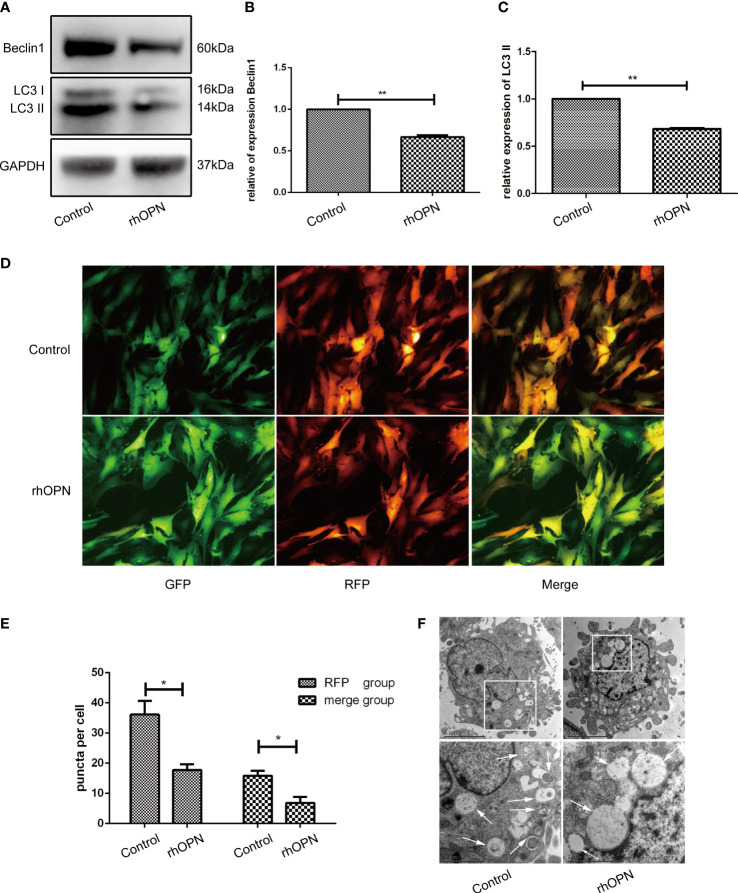
**(A)** Beclin1 and LC3 (both 3 repetitions) were significantly decreased after treatment with rhOPN in chondrocytes, as shown by western blot analysis; **(B)** Difference in expression of LC3II between the control and rhOPN groups was statistically significant (P = 0.0098, P < 0.05, **means P < 0.01); **(C)** The expression of beclin1 between the control and the rhOPN groups was statistically significant (P = 0.0049, P < 0.05, **means P < 0.01); **(D)** mRFP-GFP-LC3 puncta confirmed the inhibitory effects of rhOPN on LC3 II; **(E)** Difference in expression of LC3 between the merge and RFP groups and between the control and rhOPN groups was statistically significant (*P* = 0.0194 and *P* = 0.0268, respectively, both P<0.05, * means P<0.05). **(F)** TEM analysis of autophagosomes in chondrocytes under different conditions. Autophagosomes were marked with white arrows. Scale bar: 5 µm.

Meanwhile, the rhOPN suppressed the LC3 and Beclin1 protein after incubation with rhOPN for 48 h. The expression level of autophagy marker protein LC3II was significantly decreased after treatment with rhOPN in chondrocytes (0.68 ± 0.01-fold), and the difference between the control and the rhOPN treated group was statistically significant (*P*=0.0098, *P*<0.05), as shown in **Figures 2A, B**. Moreover, the Beclin1 protein also decreased between these two groups (0.67 ± 0.02-fold), and the difference was statistically significant difference (*P*=0.0049, *P*<0.05), as shown in **Figures 2A–C**. The mRFP-GFP-LC3 puncta also indicated that rhOPN could inhibit the autophagy in chondrocytes, as shown in **Figure 2D**, and the difference between the two groups was statistically significant (*P*=0.0194 and *P*=0.0268 respectively, both *P*<0.05), as shown in **Figure 2E**. In **Figure 2F**, the transmission electron microscopy (TEM) also confirmed that the autophagosomes were less after the rhOPN treatment than in the control.

### OPN receptors involved in OPN-induced autophagy repression

**Figure 3:**
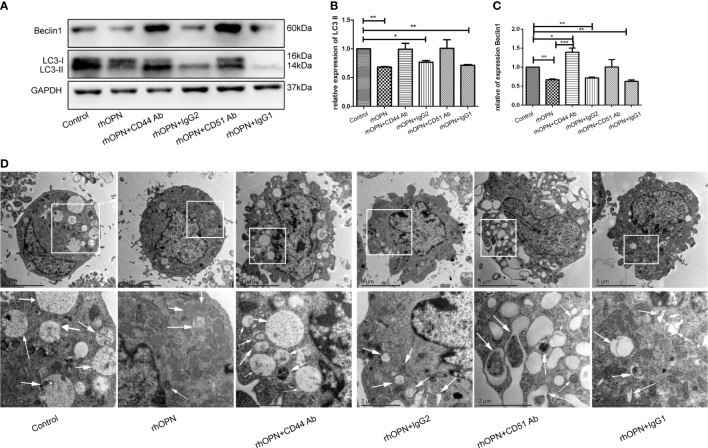
**(A)** Expression of Beclin1 and LC3 (both 3 repetitions) after each treatment, as determined by western blot analysis; **(B)** Expression of LC3 in each group; *indicates P < 0.05, **indicates *P* < 0.01, ***indicates *P* < 0.001 for the comparisons between the two groups; **(C)** Expression of Beclin1 in each group; *indicates *P* < 0.05, **indicates *P* < 0.01, ***indicates *P* < 0.001 for comparisons between the two groups. **(D)** The autophagosomes pointed by white arrows were shown by TEM images in chondrocytes.

Subsequently, to determine whether the main receptors of OPN, such as CD44 and αvβ3 integrin, were involved in the process, we used a neutralizing antibody to block receptor binding to OPN. Our results showed that prior to administration, the neutralizing antibody could block receptor binding to OPN partly, as shown in **Figures 3A–C**. The results of each group were as follows: the expression of LC3 and Beclin1 in the control group was equivalent to 1; the expression of LC3 and Beclin1 in rhOPN group was 0.68 ± 0.01-fold and 0.67 ± 0.02-fold, respectively; the expression of LC3 and Beclin1 in rhOPN+CD44 Ab group was 0.99 ± 0.08-fold and 1.39 ± 0.09-fold, respectively; the expression of LC3 and Beclin1 in rhOPN+ Ig G2 group was 0.77 ± 0.02-fold and 0.72 ± 0.02-fold, respectively; the expression of LC3 and Beclin1 in rhOPN+CD51 Ab group was 1.01 ± 0.12-fold and 1.01 ± 0.16-fold, respectively; the expression of LC3 and Beclin1 in rhOPN+ Ig G1 group were 0.71 ± 0.01-fold and 0.62 ± 0.03-fold, respectively. The expression of LC3 and Beclin1 in the rhOPN group was significantly lower than that in the control group (*P*=0.0098, *P*=0.0049, *P*<0.05), and the result also revealed that the CD44 Ab played an important role in assisting OPN in inhibiting the expression of the autophagy marker proteins Beclin1 and LC3. We also confirmed that the OPN receptor was involved in this process. The expressions of LC3 and Beclin1 were much lower in the type IgG2 group than in the control group, and the difference was significant (*P*=0.0245, *P*=0.0067, *P*<0.05). In **Figure 3D**, TEM determined the autophagosomes also proved the above changes. Taken together, our results indicated that OPN could suppress the relative expression of LC3II and Beclin1 *via* integrin and CD44 in chondrocytes.

### Signaling mechanisms involved in OPN-induced autophagy repression

**Figure 4:**
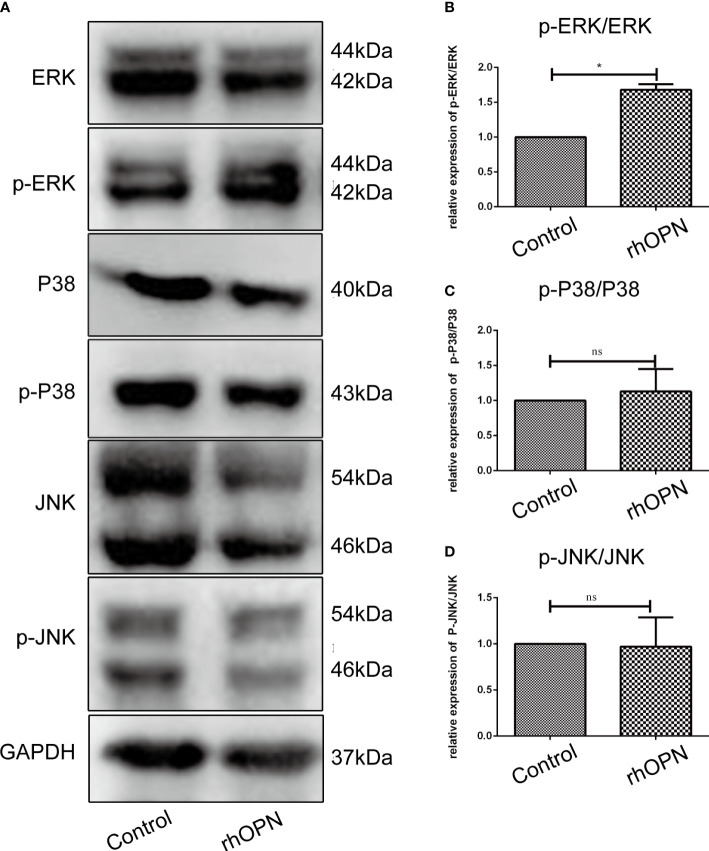
**(A)** Expression of ERK/p-ERK, P38/p-P38, and JNK/p-JNK between the control and rhOPN groups by western blot analysis; **(B)** Relative expression of p-ERK/ERK between the control and rhOPN groups (n = 3); *P* = 0.1049; *indicates a significant difference between the two groups (*P* < 0.05); **(C)** Relative expression of p-P38/P38 between the control and rhOPN groups; (n = 3) ns indicates a lack of significant differences between the two groups (*P* > 0.05); **(D)** Relative expression of p-JNK/JNK between the control and rhOPN groups (n = 3); ns indicates a lack of significant differences between the two groups (*P* > 0.05).

**Figure 5:**
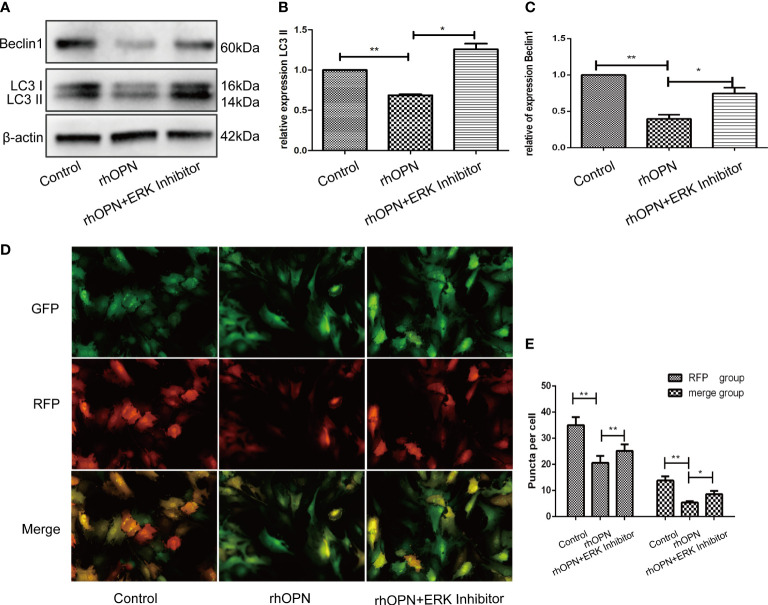
**(A)** Expression of beclin1 and LC3 among the control group, rhOPN group and ERK Inhibitor+ rhOPN group (n = 3); *indicates a significant difference in comparisons between the two groups (*P* < 0.05); **(B)** Relative expression of LC3II among each group (n = 3); P = 0.0015 for the comparison between the control group and rhOPN group; *P* = 0.0104 for the comparison between the rhOPN group and rhOPN+ERK inhibitor group; *indicates *P* < 0.05, **indicates *P* < 0.01 for the comparisons between the two groups; **(C)** Relative expression of Beclin1 among each group (n = 3); *P* = 0.0091 for comparison between the control group and rhOPN group; *P* = 0.0229 for the comparison between the rhOPN group and rhOPN+ERK inhibitor group; *indicates *P* < 0.05, **indicates *P* < 0.01 for the comparisons between the two groups. **(D)** mRFP-GFP-LC3 puncta confirmed the inhibitory effects of rhOPN and rhOPN + ERK Inhibitor reversed the inhibition on LC3 II; **(E)** Difference in expression of LC3 in the RFP groups of control vs rhOPN and rhOPN vs rhOPN + ERK Inhibitor were statistically significant (*P* = 0.0043, *P* = 0.0048 respectively, all of them *P* < 0.05, *means *P* < 0.05, **indicates *P* < 0.01); the difference in expression of LC3 in the merge groups of control vs rhOPN and rhOPN vs rhOPN + ERK Inhibitor were statistically significant (*P* = 0.0013 and *P* = 0.0406 respectively, all of them *P* < 0.05, *means *P* < 0.05, **indicates *P* < 0.01).

The MAPK pathway related proteins were also tested, and our results indicated that compared with the control group, the ratio of p-ERK/ERK was increased after the rhOPN treatment (*P*=0.0134, *P*<0.05), as shown in **Figures 4A, B**; however, the ratios of p-P38/P38 and p-JNK/JNK showed no significant difference between the rhOPN group and the control group (*P*>0.05), as shown in **Figures 4C, D**. Next, the chondrocytes were preincubated with ERK inhibitors (FR180204 10 mmol/L, Beyotime, Shanghai, China). As shown in **Figure 5A**, the activity of OPN in the suppression of autophagy was selectively inhibited by antagonists specific for ERK (FR180204). The expression of LC3II protein was greatly decreased after treatment with rhOPN in chondrocytes (0.69 ± 0.01-fold), and the difference between the control and rhOPN-treated groups was statistically significant (*P*=0.0015, *P*<0.05). While these trends could be reversed after treatment of ERK inhibitor (1.26 ± 0.06-fold), the difference between the rhOPN group and ERK inhibitor group was statistically significant (*P*=0.0104, *P*<0.05), as shown in **Figure 5B**. The Beclin1 protein also showed a similar trend as the LC3II between the control and rhOPN treated group in chondrocytes (0.40 ± 0.05-fold), and the difference was significant (*P*=0.0091, *P*<0.05). However, the trend could be reversed after the treatment of ERK inhibitor (0.40 ± 0.05-fold), there also exist a statistically significant difference (*P*=0.0229, *P*<0.05), as shown in **Figure 5C**. The mRFP-GFP-LC3 puncta also indicated that ERK inhibitor could block the decreasing effects of rhOPN on autophagy in chondrocytes, as shown in **Figures 5D, E**.

### RhOPN promoted the cellular activity of chondrocytes

**Figure 6:**
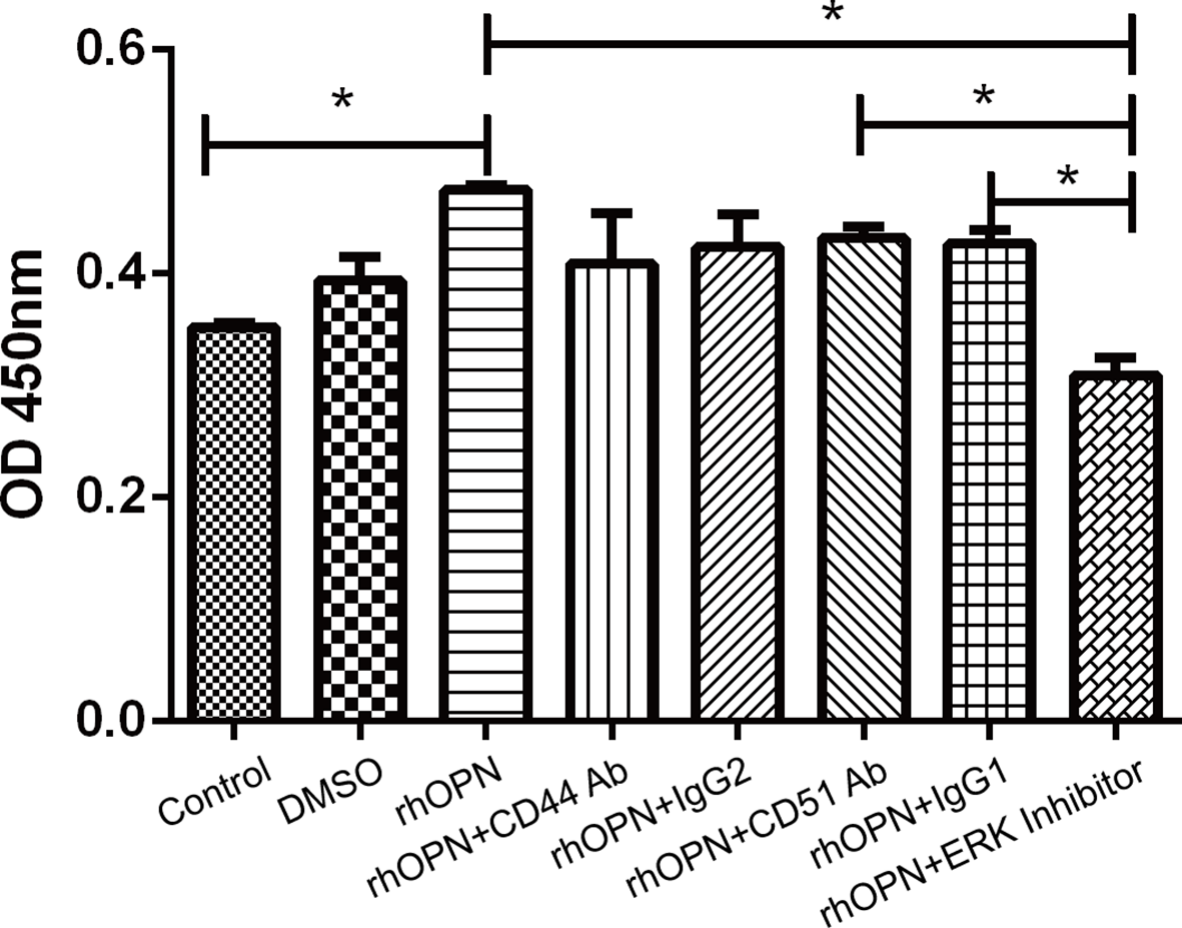
rhOPN promoted chondrocyte proliferation *in vitro*, and the ERK inhibitor blocked the effect of the Cell Counting Kit-8 assay (n = 3); *indicates *P* < 0.05 for the comparisons between the two groups.

We performed a cell proliferation assay and found that the rhOPN could greatly promote the proliferation of chondrocytes. Significant differences were not observed between the DMSO group (OD 450 nm=0.3933 ± 0.0375) and the control group (OD 450 nm =0.352 ± 0.0052) (*P*>0.05); however, the rhOPN treated group (OD 450 nm=0.461 ± 0.03032) could upregulate the chondrocytes proliferation and viability compared to the control group (OD 450 nm=0.352 ± 0.0052) (*P*=0.0172, *P*<0.05). Furthermore, after the blocking treatment with anti-CD44 Ab (OD 450 nm=0.4083 ± 0.0775) and anti-CD51/61 Ab (OD 450 nm=0.4313 ± 0.018) or their isotype IgG2 (OD 450 nm=0.4233 ± 0.0508) and IgG1 (OD 450 nm=0.4263 ± 0.0208), their OD 450 nm values did not show a significant difference. The OPN-induced proliferation of chondrocytes was significantly reduced by treatment of ERK MAPK pathway inhibitors, as shown in **Figure 6**. The OD 450nm values between rhOPN and the ERK inhibitor (OD 450 nm=0.3087 ± 0.0268) are exhibited a statistically significant difference (*P*=0.0358, *P*<0.05).

## Discussion

OA is a complex and multi tissue pathology disease characterized by progressive damage to the cartilage and subchondral bone, increased inflammatory factor release and decreased collagen synthesis (33). Chondrocytes are the only cells in the cartilage that play a central role in osteoarthritis pathogenesis, and chondrocytes form specific spatial patterns of articular cartilage, named the superficial layer, middle layer, and deep layer (34). These cells express and secrete various extracellular matrix (ECM) molecules, mainly collagen and proteoglycans, which are essential to the maintenance of normal expression and cartilage structure (35). However, the chondrocytes in OA are characterized by accelerated catabolic metabolism and suppressed anabolic metabolism, resulting in the secretion of more metal matrix proteins and the synthesis of less collagen and proteoglycans (34). Therefore, the chondrocyte function, metabolism, and spatial organization are correlated with the degeneration of OA (36).

OPN is produced by a variety of tissues and cells, including chondrocytes and synoviocytes, and its level in plasma and synovial tissue is increased in OA (13, 14). The levels of OPN in plasma, synovial fluid and cartilages are related to the progression of joint damage, and OPN can greatly upregulate the expression of matrix metalloproteinase-13 (MMP-13) in OA (37, 38). OPN increases the expression levels of the proinflammatory factors interleukin-6 (IL-6) and interleukin-8 (IL-8) cytokines in human OA chondrocytes, while phosphorylated OPN (p-OPN) upregulates the expression of interleukin-1β (IL-1β), IL-6, tumor necrosis factor (TNF-α), and nuclear factor-κB (NF-κB) and induces chondrocyte apoptosis (39, 40). Autophagy is a critical protective mechanism for maintaining cellular function and survival. Since autophagy plays an important role against mitochondrial dysfunction, its loss of function is linked with gradual cartilage degradation, cell death and OA (41). Activating autophagy by pharmacologic interventions may have a protective effect on cartilage degenerative processes in OA, and blocking autophagy has the opposite effect (41). Our present results indicated that OPN could inhibit autophagy in chondrocytes *in vitro*, OPN decreased beclicn1 and LC3 II protein expression, and the mRFP-GFP-LC3 immunofluorescence puncta confirmed this result. Our result is similar to a previous study showing that OPN could inhibit apoptosis and autophagy in colorectal cancer (42).

The cellular signaling molecules integrins and CD44 are involved in the severity of cartilage damage in human knee OA (16, 18). Integrins and ligand–receptor interactions between integrins play a significant role in binding to multiple extracellular ligand proteins, regulating cell differentiation and proliferation, and maintaining cartilage homeostasis in chondrocytes, while CD44 may mediate inflammatory processes; in addition, the CD44 level in articular cartilage is related to OA and rheumatoid arthritis (RA) disease degree and joint destruction (16, 20). Our results showed that OPN suppresses autophagic activity in chondrocytes by decreasing beclicn-1 and LC3 II protein expression through the binding of OPN to CD44 molecules. Previous literature reported that OPN binding to CD44 and integrin may increase inflammatory progression and joint destruction of articular cartilage in both OA and RA, and inhibiting this mechanism may be a hopeful strategy for OA therapy (16, 20, 43).

The MAPK signaling pathway has an essential effect on regulating the production and activity of many mediators of joint damage (44). It is well known that the molecules of the MAPK family involved in OA progression mainly include JNK, ERK, and p38 (44). The MAPK signaling pathway is activated by a great diversity of molecules, including growth factors, matrix proteins, G-protein coupled receptors, cytokine receptors, and integrins (22). A previous study reported that MAPK levels, including P38, JNK, and ERK, were all higher than those in normal tissue in OA (22). ERK is closely related to MMP production, and inhibiting ERK activation is sufficient to reduce the severity of OA lesions. P38 and JNK are mainly connected to the inflammatory process, and inhibiting of either p38 or JNK but not ERK was found to prevent IL-1-induced downregulation of peroxisome proliferator-activated receptor γ (PPARγ) in chondrocytes (44). Our present study showed that OPN suppresses autophagic activity in chondrocytes through the MAPK signaling pathway. We also verified that OPN suppresses autophagic activity through the interaction of OPN with MAPKs, especially the ERK protein.

**Figure 7:**
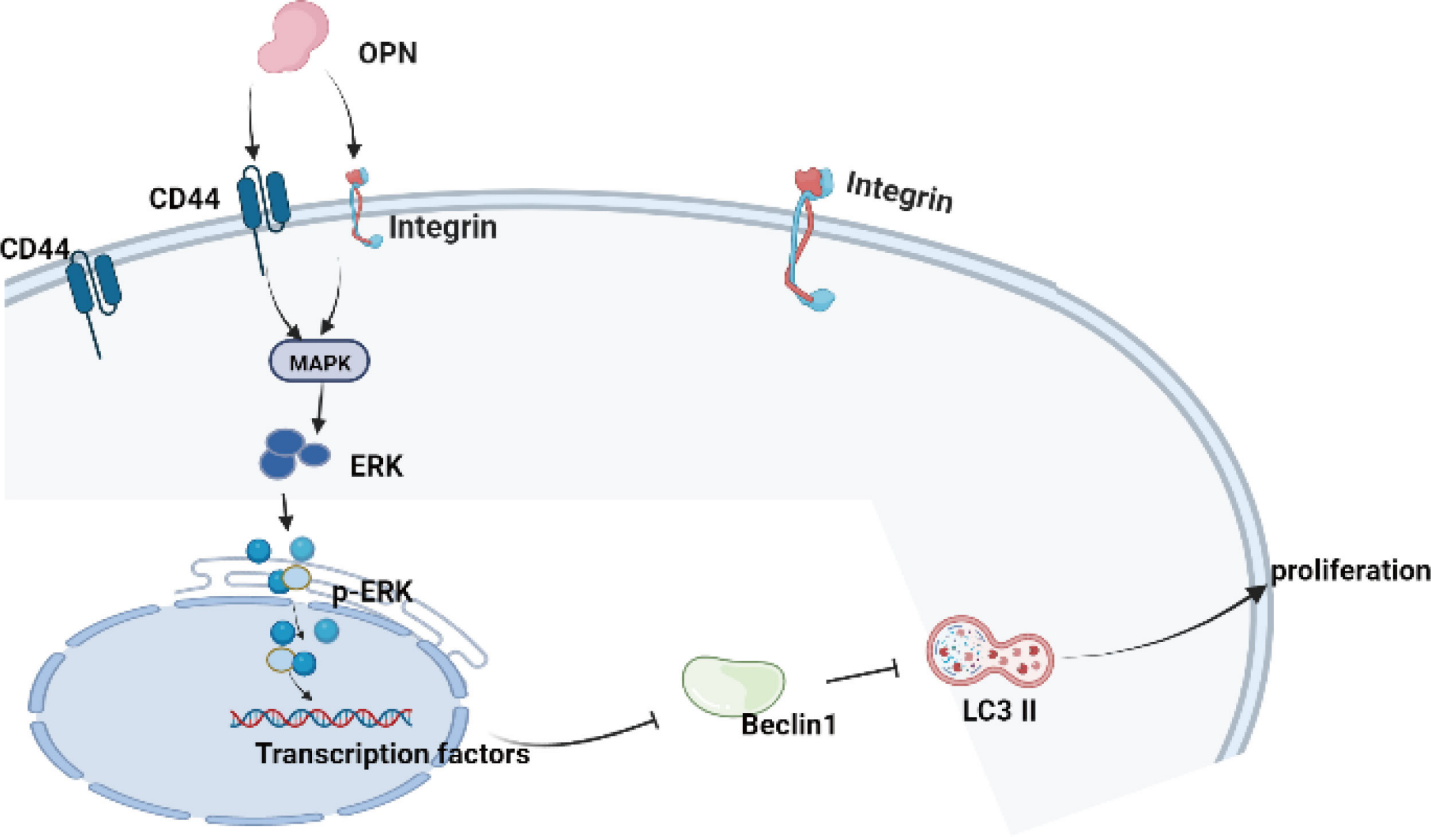
The schematic diagram of the present research indicated that OPN combined with CD44 and integrin inhibits autophagy activity through the MAPK signaling pathway in chondrocytes.

A previous study reported that chondrocyte proliferation is an important pathogenic factor of OA, and overproliferation of human chondrocytes may result in OA (45). In the OA model, rapamycin reduced the severity of cartilage degradation and decreased disintegrin-like and metallopeptidase with thrombospondin type 1 motif 5 (ADAMTS-5) expression and inflammation in synovial tissue by activating autophagy in chondrocytes (10). Our present results showed that OPN could suppress the autophagy of chondrocytes and promote the proliferation of chondrocytes, which is consistent with a previous literature report (45). Moreover, we also confirmed that OPN suppresses chondrocyte autophagy through the MAPK signaling pathway since the ERK MAPK pathway is associated with a multiple of growth factor signaling pathways that regulate cell proliferation and tissue homeostasis, and JNK and p38 are most closely related to inflammation activation and stress-induced signals (45). In summary, our results indicated that OPN suppresses the autophagy activity and promotes the proliferation of chondrocytes through the MAPK signaling pathway by binding to CD44 and decreasing the protein expression of beclicn1 and LC3 II as the **Figure 7** shown.

## Conclusion

OPN inhibited chondrocytes autophagy *via* CD44 and promoted chondrocytes proliferation through the MAPK signaling pathway in OA.

## Data availability statement

The original contributions presented in the study are included in the article/supplementary material. Further inquiries can be directed to the corresponding author.

## Ethics statement

The studies involving human participants were reviewed and approved by the Institutional Review Board of Xiangya Hospital Central South University. The patients/participants provided their written informed consent to participate in this study.

## Author contributions

R-JB, Y-SL, and F-JZ designed the study. R-JB and F-JZ wrote the manuscript. JT, D-JY, and H-ZL collected the cartilage samples and statistical analysis, DL and Y-SL cultured the primary chondrocytes and performed the mRFP-GFP-LC3 lentivirus transfer and laser confocal imaging, CCK-8 and western blots. F-JZ performed the chondrocytes immunohistochemistry staining, RJB performed transmission electron microscopy, the Y-SL and F-JZ provided the experiment cost fee. All authors contributed to the article and approved the submitted version.

## Funding

This work was supported by the National Key R&D Program of China (No.2019YFA0111900), the National Natural Science Foundation of China (No.81501923 and No. 82072506), Provincial Natural Science Foundation of Hunan (No.2020JJ3060), Administration of Traditional Chinese Medicine of Hunan Province (No.2021075), Innovation-Driven Project of Central South University (No.2020CX045), Wu Jieping Medical Foundation (No.320.6750.2020-03-14), Rui E (Ruiyi) Emergency Medical Research Special Funding Project (No.R2019007), and Independent Exploration and Innovation Project for Postgraduate Students of Central South University (No.2021zzts1037).

## Conflict of interest

The authors declare that the research was conducted in the absence of any commercial or financial relationships that could be construed as a potential conflict of interest.

## Publisher’s note

All claims expressed in this article are solely those of the authors and do not necessarily represent those of their affiliated organizations, or those of the publisher, the editors and the reviewers. Any product that may be evaluated in this article, or claim that may be made by its manufacturer, is not guaranteed or endorsed by the publisher.

## Abbreviations

Abs, Antibodies; OA, Osteoarthritis; OPN, Osteopontin; MAPK, Mitogen-activated protein kinase; MMP, matrix metalloproteinase; PBS, phosphate-buffered saline; rhOPN, human OPN; MOI, multiplicity of infection; PVDF, polyvinylidenedifluoride; JNK, Jun Nterminal kinase; CCK-8, Cell Counting Kit-8; ECM, extracellular matrix; MMP-13, matrix metalloproteinase-13; IL-6, interleukin-6; IL-8, interleukin-8; p-OPN, phosphorylated OPN; IL-1β, interleukin-1β; NF-κB, nuclear factor-κB; RA, rheumatoid arthritis; PPARγ, peroxisome proliferator-activated receptor γ; ADAMTS-5, a disintegrin-like and metallopeptidase with thrombospondin type 1 motif 5.
